# Advanced Low-Dimensional Carbon Nanomaterials for Oxygen Electrocatalysis

**DOI:** 10.3390/nano15040254

**Published:** 2025-02-07

**Authors:** Yue Yan, Ying Xin, Qingshan Zhao

**Affiliations:** State Key Laboratory of Heavy Oil Processing, College of Chemistry and Chemical Engineering, China University of Petroleum (East China), Qingdao 266580, China; 15940535308@163.com (Y.Y.); 18364538700@163.com (Y.X.)

**Keywords:** low-dimensional carbon nanomaterials, carbon dots, carbon nanotubes, graphene, oxygen reduction reaction, oxygen evolution reaction

## Abstract

Amid rising global energy demand and worsening environmental pollution, there is an urgent need for efficient energy storage and conversion technologies. Oxygen electrocatalytic reactions, specifically the oxygen reduction reaction (ORR) and the oxygen evolution reaction (OER) are critical processes in these technologies. Low-dimensional carbon nanomaterials, including zero-dimensional carbon dots, one-dimensional carbon nanotubes, and two-dimensional graphene, demonstrate substantial potential in electrocatalysis due to their unique physical and chemical properties. On the one hand, these low-dimensional carbon materials feature distinct geometric structures that enable the customization of highly active sites for oxygen electrocatalysis. On the other hand, the sp^2^ hybridization present in these materials contributes to the existence of π electrons, which enhances conductivity and facilitates catalytic activity and stability. This article reviews recent advancements in the development of efficient catalysts for oxygen electrocatalysis based on low-dimensional carbon nanomaterials, focusing on their characteristics, synthesis methods, electrocatalytic performance, and applications in energy conversion devices. Additionally, we address the current challenges faced by these nanomaterials and outline future research directions to expedite their practical applications.

## 1. Introduction

Currently, energy production and environmental pollution pose severe challenges that constrain the sustainable development of human society. The overexploitation and widespread use of fossil fuels lead to resource depletion and environmental degradation, including greenhouse gas emissions and atmospheric pollution [1]. Consequently, there is an urgent need to develop clean and efficient energy conversion technologies [2]. Among these technologies, electrochemical devices such as fuel cells, metal–air batteries [3,4], as well as hydrogen production through water electrolysis, have attracted considerable interest because of their high efficiency and eco-friendliness, with oxygen electrocatalysts serving as critical components.

**Figure 1:**
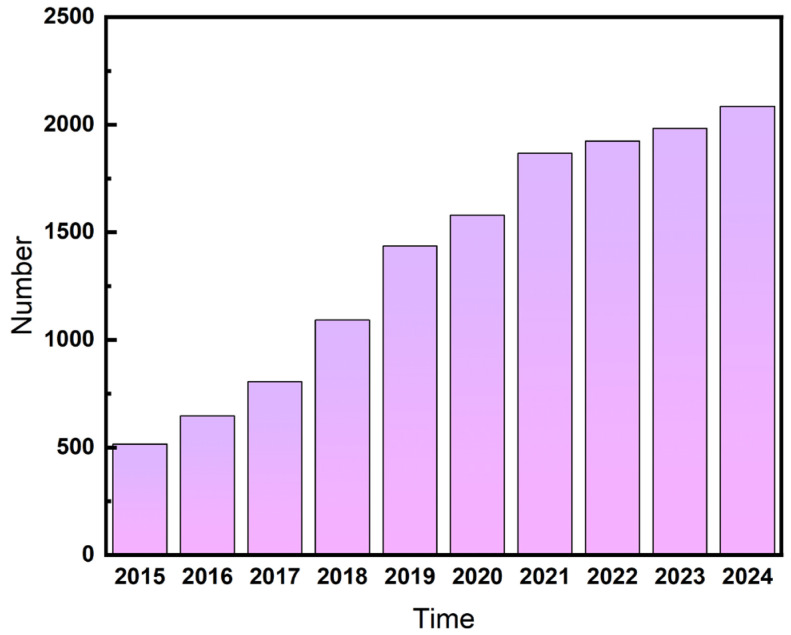
Number of research articles in the field of oxygen electrocatalysis in the last decade.

Oxygen electrocatalytic reactions, which encompass the oxygen reduction reaction (ORR) and the oxygen evolution reaction (OER) [5], are crucial for energy conversion systems such as fuel cells and water electrolysis used for hydrogen generation. High-efficiency oxygen electrocatalysts can significantly reduce the overpotential of these reactions, enhance reaction rates, and improve energy conversion efficiency, thereby boosting the performance of these devices. However, the currently prevalent noble metal-based oxygen electrocatalysts (e.g., platinum-based catalysts for ORRs and ruthenium/iridium-based catalysts for OERs) [6] face challenges such as high costs, resource scarcity, and poor stability, severely limiting their large-scale application. As a result, the pursuit of low-cost, high-performance non-noble metal-based oxygen electrocatalysts has become a prominent research focus in the energy field. The annual publication trends illustrating the growth of research in oxygen electrocatalysis over the past decade are depicted in Figure 1 below.

**Figure 2:**
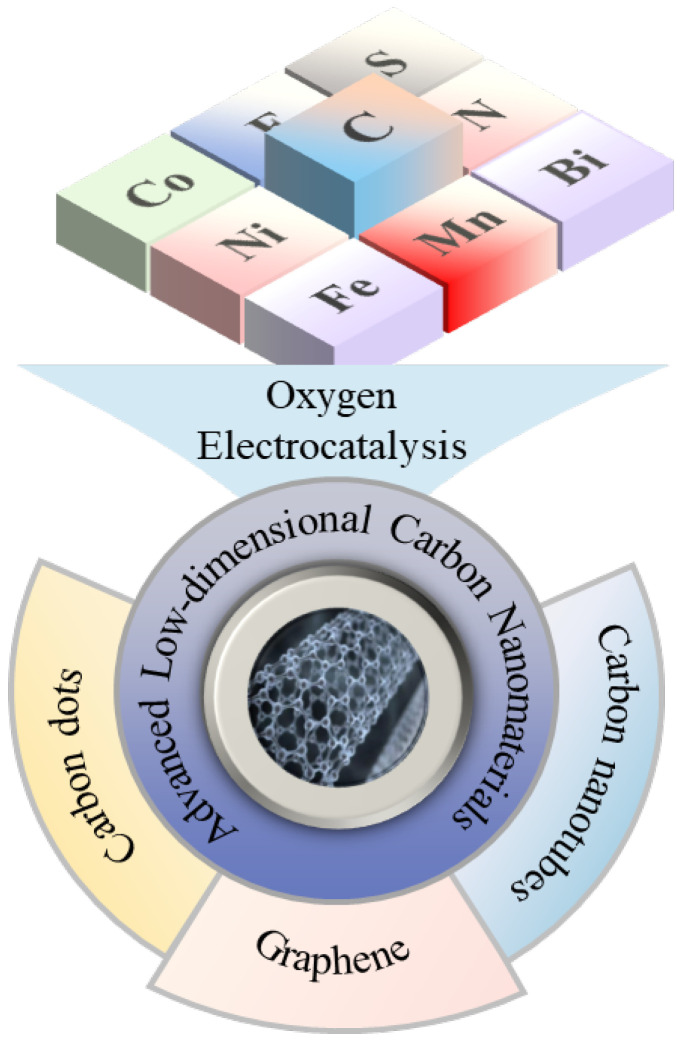
Low-dimensional carbon nanomaterial-based catalysts for oxygen electrocatalysis.

Low-dimensional carbon materials have attracted considerable attention for their electrocatalytic applications [7,8]. Materials such as zero-dimensional carbon dots (CDs), one-dimensional carbon nanotubes (CNTs), and two-dimensional graphene (Figure 2) exhibit significant potential in oxygen electrocatalysis due to their unique structures and properties. Firstly, low-dimensional carbon materials possess abundant pore structures and large specific surface areas [9], enabling the customization of highly active sites for oxygen electrocatalytic reactions. Secondly, the sp^2^ hybridization in these carbon materials contributes to the presence of π electrons, enhancing electrical conductivity and enabling quantum and edge effects, facilitating electron transfer, and enhancing reaction rates [10]. Moreover, the structure and properties of low-dimensional carbon materials are easily tunable, allowing for the introduction of other elements or functional groups through doping, surface modification, and other methods to further enhance their oxygen electrocatalytic performance. For instance, nitrogen-doped carbon materials [11] can introduce nitrogen-containing active sites into the carbon framework, improving the adsorption and activation capabilities of oxygen, and thereby enhancing catalytic activity for the ORR. Similarly, for the OER, doping carbon with heteroatoms like sulfur or phosphorus can introduce active sites [12] that enhance interactions with oxygen-related species, facilitating adsorption and activation and improving catalytic activity for more efficient oxygen evolution.

In recent years, there has been a significant increase in review articles summarizing advancements in carbon materials or transition metal-based catalysts in the field of oxygen electrocatalysis and their application in energy conversion technologies. However, a conspicuous void remains in the literature regarding comprehensive reviews that specifically address the research progress of low-dimensional carbon nanomaterials. Therefore, this review uniquely integrates recent advancements in low-dimensional carbon nanomaterials for oxygen electrocatalysis, with a particular emphasis on their characteristics, synthesis methods for electrocatalysts, electrocatalytic performance, and future challenges and directions, offering a roadmap for the development of next-generation catalysts.

## 2. Characteristics of Advanced Carbon Nanomaterials

The three classifications of low-dimensional carbon nanomaterials can be synthesized using a variety of methodologies. The specific synthesis methods and percentages, along with their respective advantages and disadvantages, are delineated in Table 1. The most commonly used method, chemical vapor deposition (CVD), accounts for 42% of applications and is favored for producing high-quality, high-purity CNTs and graphene; however, it is associated with substantial equipment costs and significant energy consumption. Conversely, it involves high equipment costs and significant energy consumption. Laser-enhanced chemical vapor deposition (LCVD, 10%) offers high production efficiency, but it requires complex and expensive setups. In addition, laser ablation (5%) is effective for synthesizing CNTs and graphene, avoiding catalyst contamination, yet it is constrained by low yield and high energy demands. Regarding the synthesis of CDs, methods like hydrothermal and solvothermal synthesis (28%) are also commonly employed, which can also be suitable for producing graphene. These techniques are lauded for their affordability, ease of implementation, and capacity to fine-tune the fluorescence properties of the CDs [13]. However, they may encounter challenges related to scalability and uniformity. Hydrothermal synthesis, in particular, is often selected for its environmentally benign characteristics and versatility, although it can be a time-consuming process. Solvothermal synthesis, while offering greater flexibility regarding solvent selection, may incur higher production costs and potentially yield less uniform particle sizes. In addition, arc discharge, microwave irradiation, self-assembly methods, etc., have also been employed for the synthesis of low-dimensional carbon nanomaterials.

### 2.1. Carbon Dots

#### 2.1.1. Characteristics of Carbon Dots (CDs)

In 2004, fluorescent carbon nanoparticles were first reported, discovered serendipitously during the purification of single-walled carbon nanotubes (CNTs) [14,15]. Two years later, in 2006, Sun et al. coined the term “carbon dots” (CDs) to describe nanoscale carbon particles that were synthesized through the laser ablation of carbon targets. CDs are remarkable zero-dimensional functional carbon-based nanomaterials known for their intrinsic fluorescence. These carbon particles are typically less than 20 nanometers in size and exhibit distinct fluorescent properties. The chemical structure of CDs can feature a hybrid of sp^2^ and sp^3^ carbon configurations, consisting of either single or multilayer graphite structures or polymer-like aggregates, which are adorned with various functional groups and polymer chains on their surfaces.

Based on variations in their carbon core microstructure, specific subclasses of CDs have emerged, including graphene quantum dots (GQDs), carbon quantum dots (CQDs), and carbonized polymer dots (CPDs). GQDs are composed of carbon cores that consist of a single layer or fewer than five layers of graphene, featuring edge bonding. These dots typically exhibit anisotropic dimensions, with their transverse size being greater than their vertical height. In contrast, CQDs generally have a spherical structure and can be classified into lattice and non-lattice carbon nanodots. CPDs are typically cross-linked flexible aggregates formed from non-conjugated polymers through dehydration and partial carbonization, lacking a defined carbon lattice structure. Additionally, a fourth type of carbon nanodots, known as carbonized nanodots (CNDs), has also been reported [16,17,18,19]. The preparation methods for CDs can be categorized into top-down and bottom-up approaches. Top-down methods involve gradually breaking down larger carbon materials into smaller CDs through physical or chemical means, including techniques such as laser ablation, arc discharge, and electrochemical methods. In contrast, bottom-up methods start with small-molecule carbon sources, such as molecules or ions, which are gradually polymerized or assembled into CDs via chemical reactions. These methods primarily include hydrothermal/solvothermal synthesis, microwave synthesis, template methods, and chemical oxidation.

As a novel addition to the carbon family, CDs demonstrate remarkable properties such as adjustable photoluminescence (PL), high quantum yield (QY), low toxicity, small dimensions, excellent biocompatibility, and availability from inexpensive sources. The structural defects inherent in CDs, along with the incorporation of heteroatoms, can generate extra electrocatalytic active sites. Additionally, the numerous functional groups on their surface and edges act as active sites or bonding locations for the development of composite or hybrid materials, facilitating the adsorption and stabilization of metal components through robust chelating interactions, which ultimately results in the creation of CD–metal composite materials. This enhances the charge transfer rate and stability of the resulting composites [20,21,22]. Additionally, the incorporation of CDs can prevent metal nanoparticles from forming ultrafine crystals with stable nanostructures, thus mitigating agglomeration and further growth of the metal nanoparticles [23].

Compared to other carbon materials, the small size of CDs provides them with exceptional electron transfer capabilities [24]. From a microscopic perspective, the nanoscale dimensions of CDs significantly shorten the electron transfer path, thereby reducing energy loss during the transfer process. This small size also induces a stronger edge quantum effect, as the proportion of edge atoms is relatively high. The chemical environment of these edge atoms differs markedly from that of the internal atoms, endowing them with high reactivity and a unique electron cloud distribution. Moreover, the extensive surface area of CDs enhances the contact points and improves the wettability between the electrode material and the electrolyte [25]. When CDs are employed as electrode materials in electrochemical systems, their extensive surface area enhances contact with the electrolyte, providing more channels and spaces for ion transport and adsorption, which facilitates efficient ion exchange. Simultaneously, good wettability ensures the even distribution of the electrolyte over the electrode surface, optimizing the interfacial environment for ion transport. This dual effect effectively reduces concentration polarization. Additionally, the high crystallinity of CDs improves electron conduction efficiency, thereby enhancing electrocatalytic performance [26]. Recent research has indicated that the intrinsic structural defects present in CDs, along with the introduction of heteroatoms, can create extra electrocatalytic active sites. Furthermore, the plentiful functional groups located on their surfaces or edges can act as active sites for the formation of composite or hybrid materials, thereby enhancing the charge transfer efficiency and stability of the composites [26]. Consequently, CDs have been widely employed to enhance the performance of ORR and OER electrocatalysis.

#### 2.1.2. CD-Based Electrocatalysts

As a novel zero-dimensional carbon material, CDs possess a unique structure and exhibit strong catalytic activity for both the ORR and OER. The small size of CDs enhances their electron transfer capabilities compared to other carbon materials and contributes to stronger edge quantum effects. Additionally, the large surface area of CDs increases the contact between electrode materials made from them and the electrolyte, which helps reduce concentration polarization and enhances ionic conductivity. The elevated crystallinity of CDs enhances their electrochemical performance, attributed to the presence of intrinsic structural defects and a significant amount of heteroatom doping. The surface and edges of CDs are rich in carbon, and their simple, modifiable structure allows for widespread use in high-efficiency OER and ORR applications.

**Figure 3:**
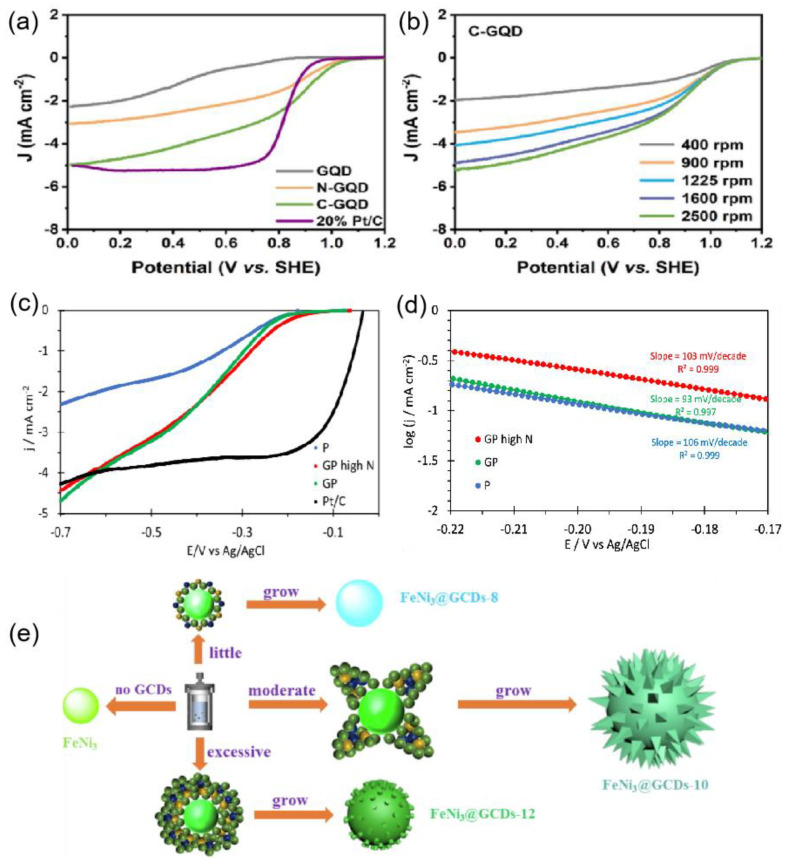
(**a**) The LSV curves of GQD, N-GQD, C-GQD, and 20% Pt/C in O_2_-saturated 0.1 M KOH solution at 1600 rpm [27]. (**b**) LSV images of C-GQD at a scan rate of 5 mV s^−1^ at different rotational speeds [27]. (**c**) Comparison at 1500 rpm [29]. (**d**) Tafel plots from LSV curves at 1500 rpm: P (black), GP high N (red), GP (magenta) [29]. (**e**) Schematic illustration for the various morphology formations of FeNi_3_ by adding GCDs with different concentrations [30].

Research has shown that metal-doped CDs can polarize the bonded carbon atoms, making the doped atom sites more favorable for oxygen adsorption and reducing the dissociation energy of oxygen. Consequently, CDs doped with heteroatoms demonstrate enhanced catalytic activities for both the OER and ORR. Sun et al. [27] proposed the synthesis of fluoro-nitrogen co-doped graphene quantum dots (C-GQDs) as a high-efficiency catalyst for the ORR. The introduction of fluorine and nitrogen atoms modulated the electronic structure of the carbon atoms, synergistically enhancing the catalytic performance. Electrochemical studies indicated that, compared to GQDs and nitrogen-doped GQDs (N-GQDs), the initial potential of C-GQDs positively shifted by 200 mV and 170 mV, respectively, while the half-wave potential positively shifted by 200 mV and 180 mV (Figure 3a), achieving performances comparable to that of 20% Pt/C catalysts (Figure 3b). In addition, Mahato et al. [28] prepared sulfur and nitrogen co-doped GQDs and TiO_2_ composites (S,N-GQDs/TiO_2_/C-800). The results demonstrated that S,N-GQDs/TiO_2_/C-800 exhibited excellent ORR activity, which can be attributed to the strong interaction between S,N-GQDs/TiO_2_ and the carbon support. This unique structure offered outstanding electrical conductivity, a high specific surface area, and efficient charge transfer kinetics between the doped GQD and TiO_2_ interfaces, facilitating the subsequent charge transfer from the carbon surface to S,N-GQDs/TiO_2_. Moreover, Suanto et al. [29] investigated nanocomposites containing boron and nitrogen co-doped CDs and anion-exchange ionomers (PO-LCs) based on poly (2,6-dimethyl polyphenyl ether) with trimethylammonium groups. The results revealed that samples with the highest nitrogen content exhibited the best electrocatalytic performance (Figure 3c), achieving an initial potential of 0.92 V, which is the highest reported for carbon-based materials, as well as a current density of 4.5 mA cm^−2^ at −0.7 V and 1500 rpm (Figure 3d).

CDs can modify the morphology and conductivity of catalysts to enhance their OER electrocatalytic performance. Li et al. [30] proposed the introduction of GCDs to modify the conductivity and morphology of the FeNi_3_ alloy (Figure 3e). The expanded active area, significantly improved conductivity, and the strong synergistic coupling effect resulted in the optimized FeNi_3_@GCDs-10 catalyst exhibiting an overpotential of 238 mV at a current density of 10 mA cm^−2^, accompanied by a Tafel slope of 48.7 mV dec^−1^. These metrics not only underscore the catalyst’s effectiveness in enhancing reaction kinetics but also highlight the potential of CDs to transform conventional catalysts into high-performance materials. Similarly, Zhu et al. [31] developed an iridium-based oxide catalyst with a heterogeneous phase (h-IrO_2_@CDs) by incorporating CDs for modification. This approach altered its morphology and enhanced its conductivity, with the CDs effectively bridging the 1T phase and rutile phase within the heterogeneous iridium-based oxide catalyst, creating a structure that offers additional active sites and improves the catalyst’s conductivity. As a result, the catalyst demonstrated excellent OER catalytic performance and stability in acidic environments, achieving a low overpotential of 161 mV at 10 mA/cm^2^.

**Figure 4:**
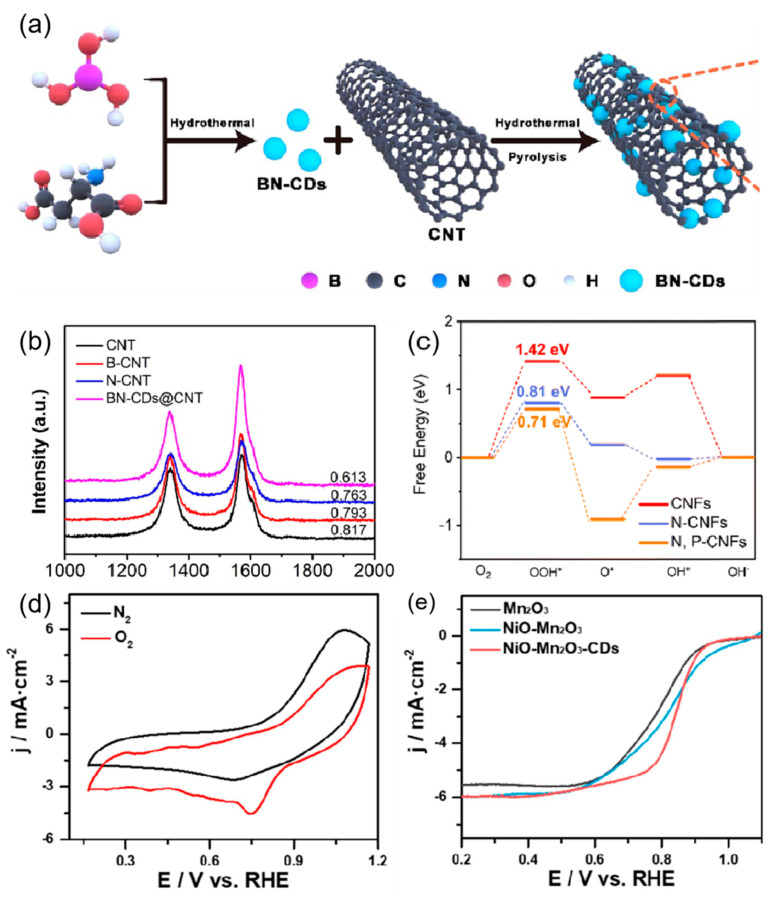
(**a**) Schematic illustration of the synthesis of the BN-CDs@CNT and (**b**) Raman spectra [32]. (**c**) Free energy diagram for the ORR pathway of different sites of CNFs, N-CNFs and N,P-CNFs under alkaline conditions [33]. (**d**) CV curves of NiO-Mn_2_O_3_-CDs in N_2_ and O_2_-saturated 0.1 M KOH with a scan rate of 0.1 V·s^−1^ [34]. (**e**) ORR polarization curves of Mn_2_O_3_, NiO-Mn_2_O_3_ and NiO-Mn_2_O_3_-CDs at 1600 rpm in O_2_-saturated 0.1 M KOH [34].

CDs can also increase active sites through a synergistic effect with other substances, thus exhibiting good electrocatalytic activity in both the OER and ORR. Pei et al. [32] combined boron and nitrogen co-doped CDs with multi-walled carbon nanotubes (MWCNTs) to prepare three-dimensional nanocatalysts (Figure 4a). The catalyst exhibited high electrical conductivity and a large specific surface area, similar to MWCNTs while exposing numerous CD-rich marginal active sites. This unique feature enhanced electron transfer, resulting in excellent performance in the ORR (Figure 4b). Moreover, Yuan et al. [33] created an effective catalyst for the ORR by combining carbon nanofibers (PCNFs) that were co-doped with nitrogen and phosphorus with CDs. These PCNFs were characterized by a well-defined pore architecture and a large specific surface area, with nitrogen and phosphorus heteroatoms evenly distributed within the catalyst. The addition of CDs altered the electronic properties of PCNFs by introducing heteroatoms and defect sites, reducing free energy, and modifying the adsorption energy of oxygen (Figure 4c). In addition, Shao et al. [34] bridged nickel oxide and manganese trioxide via oxygen-containing groups (NiO-Mn_2_O_3_-CDs) for the ORR and OER. Experimental results demonstrated that NiO-Mn_2_O_3_-CDs exhibited remarkable electrocatalytic performance, characterized by a low overpotential of 298 mV, leading to an OER current density of 10 mA cm^−2^ and a high half-wave potential of 0.84 V (Figure 4d,e). The covalent bridging of CDs with nickel and manganese atoms altered the electronic structure of the active site and enhanced charge transfer, resulting in excellent OER and ORR electrocatalytic properties.

### 2.2. Carbon Nanotubes

#### 2.2.1. Characteristics of Carbon Nanotubes (CNT_S_)

Carbon nanotubes (CNTs) are a type of carbon characterized by a diameter in the nanometer range and a length in the micrometer range, resulting in a length-to-diameter ratio that exceeds 1000 [35]. The atoms in CNTs are arranged hexagonally, similar to the structure of graphite. Specifically, CNTs consist of rolled cylindrical sheets of graphite, known as graphene, which form seamless cylinders with diameters in the order of nanometers [36]. CNTs can have open ends or be capped with fullerene-like structures. Their name reflects their dimensions, as CNTs measure only a few nanometers in diameter—approximately 50,000 times narrower than a human hair—while their lengths can reach several micrometers.

CNTs can be categorized according to the number of graphene layers into three types: single-walled carbon nanotubes (SWCNTs), double-walled carbon nanotubes (DWCNTs), and multi-walled carbon nanotubes (MWCNTs). SWCNTs are typically narrower, with diameters ranging from 1 to 2 nanometers, and they tend to be curved rather than straight [37]. These tubes can be visualized as seamless cylinders created by rolling up a single layer of graphite (a graphene layer). SWCNTs comprise two distinct regions with varying physical and chemical properties: the side wall and the end cap of the tube [38]. In contrast, MWCNTs can be viewed as collections of concentric SWCNTs with varying diameters, formed by multiple layers of rolled graphite.

Due to their structural characteristics, CNTs are considered nearly one-dimensional [36]. The bonds within CNTs are predominantly sp^2^-hybridized, forming a honeycomb lattice structure in which each carbon atom is bonded to three neighboring atoms, similar to the bonding in graphite. This bond structure is stronger than the sp^3^ bonds found in diamonds, contributing to the exceptional strength of CNTs. The CNTs naturally aggregate into bundles due to van der Waals forces. When subjected to high pressure, CNTs can merge, leading to the exchange of some sp^2^ bonds for sp^3^ bonds, which allows for the potential creation of strong, infinitely long wires through high-pressure connections. Overall, CNTs possess a nanoscale hollow tubular structure formed by curling the layered structure of graphite [39].

The unique organizational structure of CNTs imparts several excellent properties, including a high length-to-diameter ratio, large surface area, excellent electrical conductivity, good plastic toughness, and high mechanical strength [40]. These attributes have led to significant demand for CNTs in both commercial and industrial applications [41,42]. Due to their unique structural advantages, such as rapid electron and mass transfer, low aggregation, and high solubility, CNTs are ideal candidates for enhancing the catalytic activity of the ORR and OER [43]. Furthermore, as a rolled sheet of graphite, CNTs exhibit excellent intrinsic properties, including heat resistance, corrosion resistance, thermal shock resistance, good heat transfer and electrical conductivity, self-lubrication, and biocompatibility [44]. The scale, structure, and atomic arrangement of CNTs confer them with unique advantages.

Firstly, the nanoscale microstructure of CNTs, with diameters at the nanometer level and lengths reaching several micrometers to millimeters, yields a large aspect ratio, making them quasi-one-dimensional quantum wires. This one-dimensional hollow structure can serve as a template for synthesizing other one-dimensional nanostructured materials through filling, wrapping, and space-limiting reactions [45,46,47]. Secondly, CNTs possess special electrical properties [48,49]. For certain types of CNTs, the valence band and conduction band overlap, resulting in a partially filled band that allows electrons to move freely, exhibiting metal-like conductivity. The excellent electrical conductivity of CNTs arises from sp^2^ hybridization, where each carbon atom has an unpaired electron located in the π orbital, perpendicular to the layered structure [50,51]. The conductivity of CNTs is influenced by their diameter and the helical angle of the tube wall; when the diameter exceeds 6 nm, electrical conductivity typically decreases. Depending on their configuration, CNTs can exhibit either metallic or semiconducting properties. Additionally, CNTs demonstrate exceptional mechanical properties [52,53,54,55]. Composed of C=C covalent bonds formed by sp^2^ hybridization—some of the strongest bonds in nature—CNTs are among the strongest and most rigid materials known. Although their structure resembles that of polymer materials, CNTs are significantly more stable. They currently represent the materials with the highest specific strength available. When combined with other engineering materials to form composite materials, CNTs enhance the strength, fatigue resistance, elasticity, and isotropy of the composites, considerably improving their overall properties [56,57,58,59]. Finally, CNTs exhibit excellent heat transfer performance [60,61]. Their large length-to-diameter ratio facilitates high heat transfer efficiency along their length, while heat transfer performance in the perpendicular direction is comparatively lower. By optimizing orientation, CNTs can be synthesized into highly anisotropic thermal conduction materials. Moreover, the incorporation of even a small amount of CNTs into composite materials significantly enhances their thermal conductivity [62,63].

#### 2.2.2. CNT-Based Electrocatalysts

CNTs possess unique structural advantages, including rapid electron and mass transfer, a large surface area, low aggregation, and high solubility. These characteristics make CNTs ideal candidates for enhancing the catalytic activity of the ORR and OER, leading to their widespread use in improving ORR/OER performance. However, pristine carbon nanotubes are often too inert for effective ORR/OER catalysis. To address this limitation, heteroatom doping—similar to approaches used with CDs and graphene—is frequently employed to modify their surface properties. In particular, nitrogen-doped carbon nanotubes have garnered significant attention [64,65], with pioneering work by L. Dai et al. [66] demonstrating that vertically arranged nitrogen-containing CNTs can exhibit greater catalytic activity and stability in comparison to conventional Pt/C catalysts.

**Figure 5:**
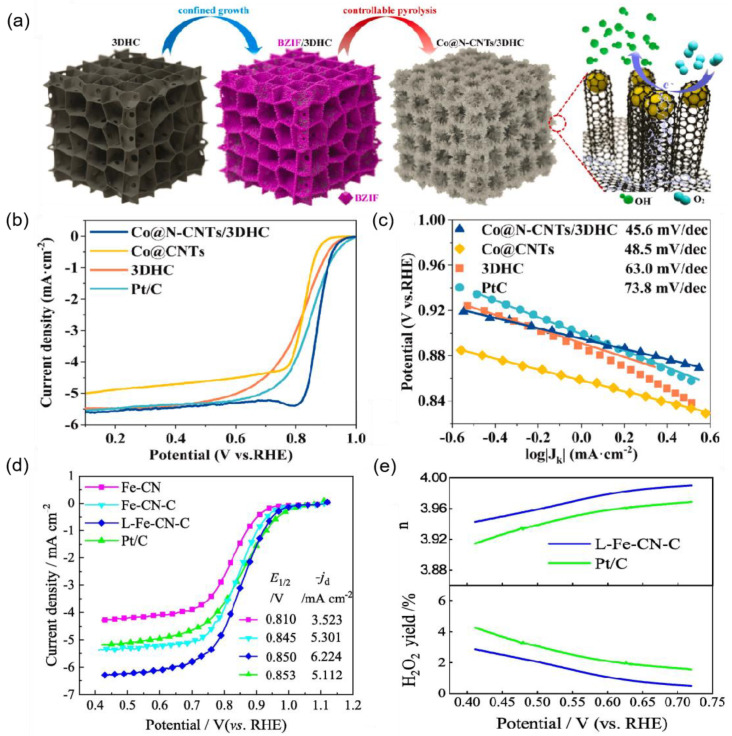
(**a**) The preparation illustration of Co@N-CNTs/3DHC electrocatalyst [67]. (**b**) Electrochemical ORR performance of Co@N-CNTs/3DHC evaluated at 0.1 M KOH. LSV curves with rotation speed of 1600 rpm [67]. (**c**) Electrochemical ORR performance of Co@N-CNTs/3DHC evaluated at 0.1 M KOH. Tafel plots [67]. (**d**) LSV curve of various catalysts to ORR and (**e**) H_2_O_2_ yield and electron transfer number [64].

**Figure 6:**
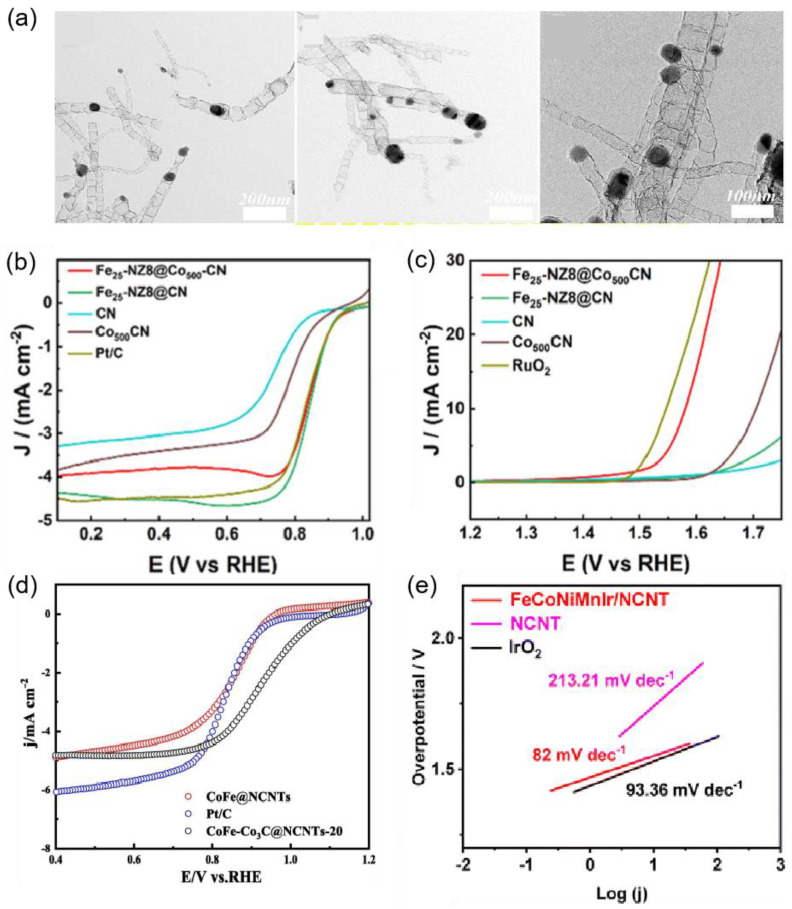
(**a**) TEM images of CoFe-Co_3_C@NCNTs-20 [71]. (**b**) LSV curves of the above various catalysts [70]. (**c**) LSV curves for the OER [70], and (**d**) LSV curves of CoFe-Co_3_C@NCNTs-20 [71]. (**e**) Tafel slope of FeCoNiMnIr/NCNT, IrO_2,_ and NCNT [72].

Numerous studies have shown that doping CNTs with various elements can enhance their ORR and OER properties. The carbon atoms neighboring the nitrogen dopants display a significant positive charge density, making them active sites for catalysis. For instance, Tang’s team [67] developed a layered electrocatalyst for the ORR, named Co@N-CNTs/3DHC, featuring ultrafine cobalt nanoparticles embedded in nitrogen-doped CNTs, which are securely fixed within a three-dimensional cellular porous carbon (3DHC) nanochamber (Figure 5a). The synthesis strategy involved constructing a precursor and controlling the pyrolysis step, with detailed analysis revealing the evolutionary mechanism of Co@N-CNTs/3DHC based on precursor gasses and carbonized products. Driven by electron reconstruction, the resulting lamellar Co@N-CNTs/3DHC demonstrated impressive ORR activity, achieving a half-wave potential of 0.88 V in alkaline media (Figure 5b,c). He’s team [68] developed a simple one-step synthesis method for nitrogen and phosphorus co-doped CNTs (N/P-CNTs) using ammonium dihydrogen phosphate and 2-methylimidazole as precursors, with CNTs serving as the matrix material. The resulting N/P-CNT-900 demonstrated commendable catalytic activity for the ORR in alkaline environments, with an E_1/2_ of 0.83 V, along with impressive methanol tolerance and stability. Additionally, catalysts for zinc–air battery (ZAB) structures are advantageous for use in electrochemical energy conversion devices, providing pathways for the development of inorganic metal ORR catalysts. Xu et al. [69] synthesized nitrogen and sulfur co-doped carbon nanotubes (CNTs-NS) featuring a high density of defects by employing a novel method that combines molecular-level co-doping with lattice defect construction. The synergistic effects of surface defects and N and S diatomic co-doping resulted in CNTs-NS exhibiting outstanding performance in OER and ORR electrocatalysis, emphasizing the importance of structural defects in enhancing catalytic activity. In another study in 2023, Lei et al. [64] prepared a 1D/0D composite catalyst (L-Fe-CN-C) composed of nitrogen-doped carbon nanotubes and nanoparticles, which features high space utilization, a large specific surface area, and a mesoporous microstructure. It exhibited remarkable ORR activity with an E_1/2_ of 0.850 V and a finite diffusion current density of 6.224 mA cm^−2^ (Figure 5d). The catalyst’s hydrogen selectivity toward the four-electron pathway indicates enhanced electronic efficiency (Figure 5e), showcasing its potential for practical applications in fuel cells. Similarly, Liu et al. [70] successfully doped iron and cobalt bimetals into a three-dimensional porous material coated with carbon nanotubes, creating a cost-effective bifunctional oxygen electrode material. The synthesized Fe_25_-NZ8@Co_500_-CN catalyst demonstrated high electrocatalytic activity for the ORR (E_1/2_ = 0.85 V) (Figure 6b) and OER (η_j_ = 10 = 350 mV) (Figure 6c). Moreover, Yi et al. [64] produced cobalt and nitrogen co-doped carbon nanotubes (Co/N-CNTs) through surface modification of CNTs with cobalt salt and melamine, followed by pyrolysis. This catalyst exhibited excellent ORR/OER activity with a low total potential difference (ΔE = 0.77 V), demonstrating remarkable durability.

**Figure 7:**
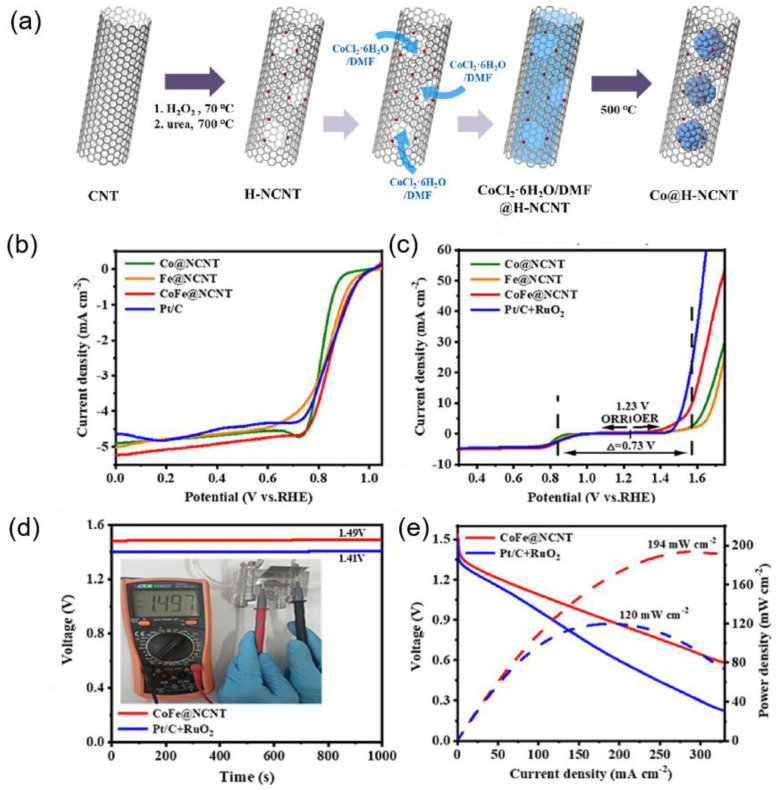
(**a**) Schematic illustration for the fabrication of Co@H-NCNT [73], and (**b**) LSV curves [74], and (**c**) bifunctional LSV curves of the contrast samples [74], and (**d**) open-circuit voltage [74], and (**e**) discharge curves during diverse current densities (1 to 50 mA cm^−2^) [74].

Heteroatom-doped carbon nanotubes loaded with metal or metal compounds are recognized as potent catalysts for the ORR and OER. These catalysts feature a unique porous structure that significantly enhances the activity of the composite catalyst by providing abundant channels for mass transfer and exposing more active sites due to their large specific surface area. Meng et al. [71] prepared a cobalt–carbide stabilized CoFe alloy (CoFe-Co_3_C@NCNT) catalyst coated with hollow bamboo nitrogen-doped CNTs, featuring a hollow bamboo-like architecture with an average diameter of approximately 50 nm (Figure 6a). The optimized CoFe-Co_3_C@NCNTs-20 catalyst displayed high ORR activity (half-wave potential of 0.934 V) (Figure 6d) and relatively good OER activity (overpotential of 0.320 V), outperforming commercial Pt/C. In addition, Pan et al. [72] fabricated high-entropy alloy (HEA) nanoparticles encapsulated in nitrogen-doped carbon nanotubes (FeCoNiMnIr/NCNTs) for the ORR and OER. The introduction of a small number of iridium atoms significantly enhanced the OER activity of the catalyst, surpassing that of commercial IrO_2_. In ORR tests, the E_1/2_ of FeCoNiMnIr/NCNT was 0.87 V (Figure 6e), demonstrating a positive shift of 17 mV compared to Pt/C, while also exhibiting excellent OER electrocatalytic performance. Moreover, Eun et al. [73] prepared porous nitrogen-doped carbon nanotubes (H-NCNTs) and subsequently encapsulated cobalt nanoparticles within the pores of H-NCNTs to form a new catalyst (Co@H-NCNT) (Figure 7a). This core–shell microstructure effectively formed catalytic active sites, enabling the electrocatalytic activities of the ORR and OER to rival those of Pt/C and IrO_2_. Similarly, Wang et al. [74] successfully encapsulated CoFe alloy nanoparticles within nitrogen-doped carbon nanotubes (CoFe@NCNTs). The Co(Fe)-N_x_ sites provided abundant active sites and synergistic interactions between the CoFe alloy and carbon nanotubes. This design not only enhanced efficient mass transfer and interfacial charge transfer but also resulted in impressive bifunctional electrocatalytic performance with a minimal potential difference (ΔE = 0.73 V) (Figure 7b,c). Such low potential differences are crucial for practical applications, indicating the catalyst’s efficiency. Further, CoFe@NCNT is specifically applied in ZABs, which shows an open-circuit voltage of 1.49 V, exceeding that of Pt/C+RuO_2_ (1.41 V) (Figure 7d). Additionally, it achieves a maximum peak power density of 194 mW cm^−2^ (Figure 7e).

### 2.3. Graphene

#### 2.3.1. Characteristics of Graphene

In 2004, Andre Geim and Konstantin Novoselov successfully isolated graphene from graphite using a seemingly simple yet highly innovative technique known as the “scotch-tape peeling method” [75]. Since then, two-dimensional (2D) materials have attracted significant scientific interest [76]. Graphene, consisting of a single layer of carbon atoms arranged in an sp^2^-bonded aromatic lattice, has been at the forefront of scientific research and practical applications for the past decade [77,78]. The carbon atoms in graphene are tightly arranged in a hexagonal honeycomb lattice, where each carbon atom forms three σ-bonds with three neighboring carbon atoms. This bonding configuration results in an exceptionally flat two-dimensional planar structure, contributing to graphene’s remarkable mechanical strength and unique electronic properties. Furthermore, its thickness is only one atomic layer—approximately 0.335 nm—making it the thinnest known material, which contributes to its unique two-dimensional properties.

As a single plane of graphite, graphene exhibits a multitude of intriguing and exceptional properties. It possesses a high theoretical specific surface area (~2630 m^2^ g^−1^) [79,80], remarkable charge carrier mobility (~100,000 cm^2^ V^−1^ s^−1^) [81], excellent white light transmittance (~97.7%) [82], exceptional mechanical strength (with a Young’s modulus of 1 TPa) [58], and superior thermal conductivity (ranging from 3000 to 5000 W m^−1^ K^−1^) [83]. These unique characteristics make graphene an attractive material for various applications in electronic devices, photovoltaic systems, heterogeneous catalysis, and more. Significant efforts have been made to develop synthesis methods for graphene and its derivatives. Currently, high-quality graphene can be obtained through techniques such as chemical vapor deposition (CVD), epitaxial growth, mechanical exfoliation, and liquid-phase exfoliation [84]. Among these, the chemical oxidation–reduction method has become the most popular approach due to its lower cost and scalability. This method involves oxidizing graphite to produce graphene oxide (GO) [85], which is then reduced to obtain reduced graphene oxide (RGO). GO serves as a fundamental substrate for constructing graphene-based derivatives or composites. Although the oxygen-containing groups (primarily carboxyl, hydroxyl, and epoxy) [86] in GO cannot be completely removed—resulting in altered physicochemical properties—the excellent dispersibility, ease of modification, and adsorptive and catalytic abilities of GO ensure its extensive practical applications.

Compared to other carbon nanomaterials, graphene and GO possess a high specific surface area [87], making them ideal candidates for use as electrocatalytic support carriers. Their excellent electronic properties—particularly, prominent charge carrier mobility, high conductivity, and a conjugated system of large π bonds [88]—facilitate rapid electron transfer during catalytic processes. Additionally, the abundant anchoring sites on the graphene surface provide a solid foundation for the stable deposition of metal species [89], which is essential for maintaining the long-term stability and activity of catalysts. These anchoring sites arise from the unique atomic-scale structure of graphene. The planar structure exposes a significant number of carbon atoms on its surface [90], with each carbon atom’s specific bonding configuration and electronic properties allowing it to serve as an anchoring site or contribute to the formation of one.

Moreover, graphene, particularly GO, can be assembled into laminar structures through filtration or coating techniques [91], creating fast and selective 2D nanochannels for transporting small molecules or ions. These nanochannels are precisely sized at the nanoscale, typically ranging from a few nanometers to tens of nanometers in width. The size and chemical nature of the nanochannels can be finely tuned by adjusting the inter-layer spacing of the GO laminar structure. For instance, the presence of oxygen-containing functional groups on GO, such as hydroxyl, epoxy, and carboxyl groups, can influence the surface charge and hydrophilicity of the nanochannels [92]. Consequently, assembled graphene or GO membranes, with their unique structural and electronic properties, have emerged as highly promising candidates for applications in electrocatalytic reactions. These membranes possess a 2D planar structure, providing a large surface area for catalytic reactions and enabling efficient charge transfer, making them particularly suitable for applications such as the ORR and electrocatalytic water splitting.

The performance of graphene-based composites generally relies on the intricate synergistic interactions between graphene and the materials attached to the graphene nanosheets. Graphene serves as a highly conductive and stable support matrix. When other materials, such as metal nanoparticles, metal oxides, or organic polymers, are attached to the graphene nanosheets, a synergy is established. For example, metal nanoparticles deposited on graphene benefit from its high electrical conductivity, facilitating rapid electron transfer during catalytic reactions. Simultaneously, the extensive surface area of graphene provides a high-density anchoring site for the metal nanoparticles, preventing their aggregation and maintaining high catalytic activity. Thus, by rationally designing functionalization processes, it is possible to maximize the synergistic effects in graphene-based nanocomposites, leading to the development of highly efficient oxygen electrocatalysts for various energy-related applications.

#### 2.3.2. Graphene-Based Electrocatalysts

**Figure 8:**
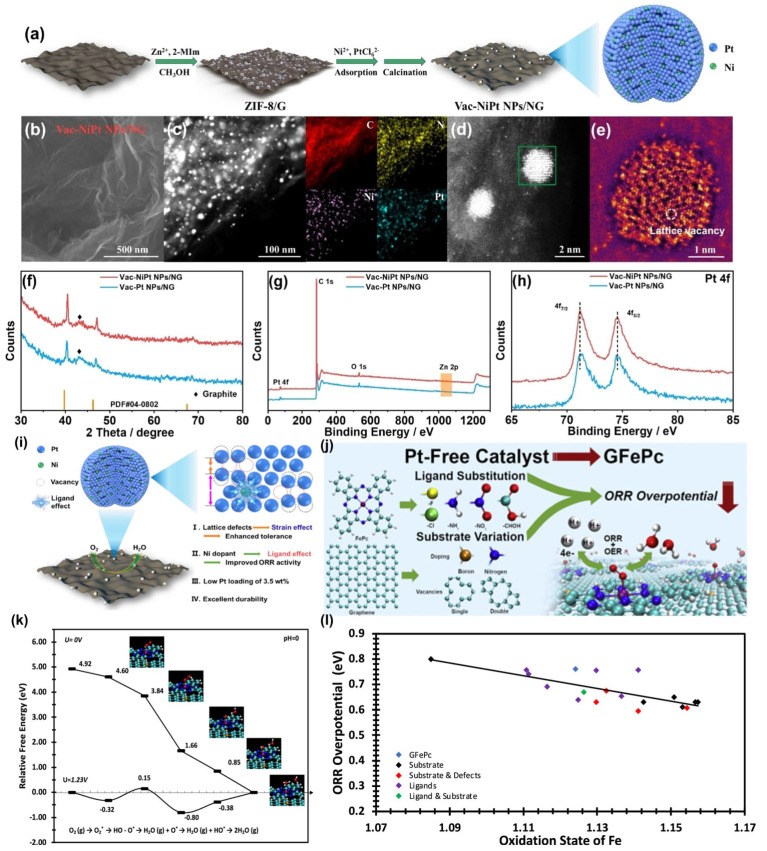
(**a**) Schematic illustration of the synthetic process of Vac-NiPt NPs/NG; (**b**) FE-SEM, (**c**) HAADF-STEM image and corresponding EDS mapping of Vac-NiPt NPs/NG; The aberration-corrected HAADF-STEM image (**d**) before and (**e**) after high-pass filtration of Vac-NiPt NP; (**f**) XRD patterns of Vac-NiPt NPs/NG and Vac-Pt NPs/NG, XPS of (**g**) survey and (**h**) Pt 4f spectra of Vac-NiPt NPs/NG and Vac-Pt NPs/NG, respectively. (**i**) Schematic diagram of the structure and mechanism of Vac-NiPt NPs/N [95]. (**j**) Structural evolution and electrochemical reaction mechanism of platinum-free catalysts. (**k**) Associative mechanism electrocatalytic reaction free-energy landscapes of ORR followed by complete WFR steps at two different electrode potentials (0 and 1.23 V) in a vacuum environment for MB+SD-GFePc. The energies are relative to two water molecules produced on the MB+SD-GFePc surface, for which a free-energy change is 4.92 eV. (**l**) Oxidation state of Fe explored as a descriptor of overpotential (eV) [94].

The surface of graphene can be functionalized with various functional groups, such as nitrogen- and oxygen-containing groups, serving as nucleation sites for the synthesis of metal single atoms or nanoparticles [93]. This capability makes graphene an ideal substrate for loading single-atom metal catalysts, significantly enhancing their electrocatalytic performance. In this context, Naomil Helse et al. [94] explored the optimization of a platinum-free catalyst for cathode fuel cells, focusing on an iron phthalocyanine monolayer supported by a graphene substrate (GFePc). They introduced vacancies into the graphene and tested a wider range of ligand exchanges to evaluate their impact on the ORR and OER. Their results indicated that a single vacancy combined with boron doping in the graphene substrate (referred to as MB+SD-GFePc) achieved the lowest ORR overpotential of 0.47 eV and a moderate OER overpotential of 0.95 eV among the modifications assessed. Furthermore, most modifications to the ligands and substrate were shown to improve ORR performance to some degree. Ye et al. [95] synthesized nickel-doped, vacancy-rich platinum nanoparticles anchored on nitrogen-doped graphene (Vac-NiPt NPs/NG) (Figure 8a), achieving a low platinum loading of 3.5 wt% and a Ni/Pt ratio of 0.038:1. Physical characterizations confirmed the presence of abundant atomic-scale vacancies within the platinum nanoparticles, which induced long-range lattice distortions. The nickel dopant facilitated a ligand effect, promoting electronic transfer from nickel to platinum (Figure 8b–e). Experimental results and theoretical calculations demonstrated that the atomic-scale vacancies enhanced the catalyst’s tolerance toward carbon monoxide (CO) and methanol (CH_3_OH). The ligand effect, stemming from the small amount of nickel dopant, accelerated the transformation from *O to *OH species, thereby improving the ORR activity without compromising tolerance capabilities (Figure 8f–h). Due to the synergistic interaction between atomic-scale vacancies and the ligand effect, the Vac-NiPt NPs/NG demonstrated improved ORR activity, good tolerance, and excellent durability. However, despite the promising results, the long-term stability of the catalyst under practical operating conditions remains a concern, as the structural integrity of vacancy-rich nanoparticles and the nickel dopant may degrade over time. Additionally, the low Ni/Pt ratio, while enhancing the ligand effect, could limit further optimization of the catalytic performance.

**Figure 9:**
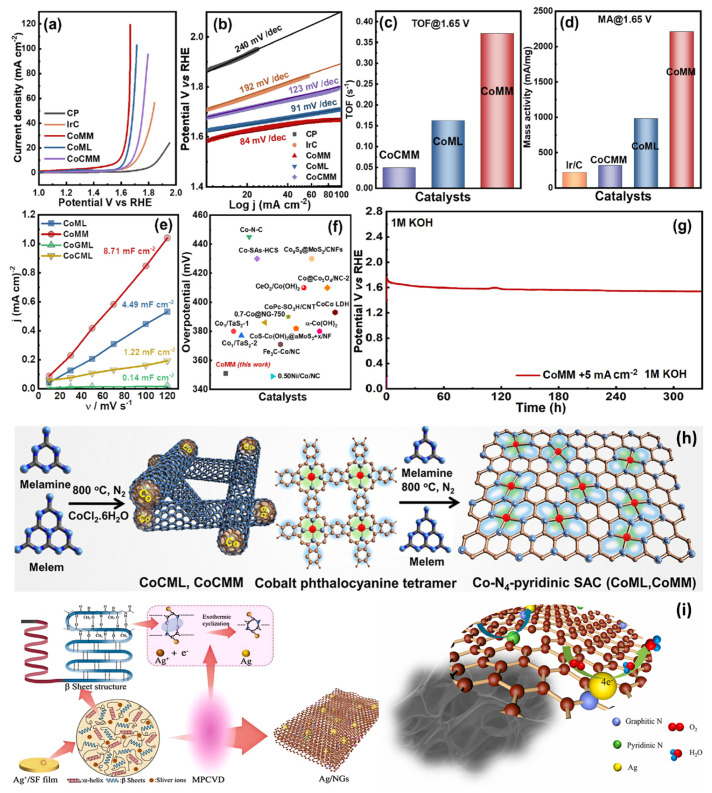
Electrocatalytic OER performance of Co-based electrodes (CP: carbon paper). (**a**) OER LSV study at 5 mV S^−1^. (**b**) Tafel slopes. (**c**) TOF at 1.65 V. (**d**) Mass activity. (**e**) Cdl values from ΔJ vs. ν in a non-Faradaic region. (**f**) Comparison of OER activities (@10 mA cm^−2^) of CoMM with other catalysts. (**g**) Long-term stability of CoMM for 300 h at 5 mA cm^−2^. Schematic diagram of the synthesis of (**h**) nanocluster CoCML and CoCMM using thermal condensation (800 °C) of melamine and melem, respectively, and Co-N_4_-pyridinic SACs using thermal condensation of cobalt phthalocyanine tetramer (CoPc) with melem (CoMM) and CoPc with melamine (CoML) [96]. (**i**) Schematic diagram of the growth mechanism of the Ag-NGs nanocomposites [97].

Graphene not only serves as a crucial substrate material but also holds potential as an active center for electrochemical reactions. Md Golam Kibria et al. [96] reported a synthesis method for single-atom catalysts assisted by macromolecules (Figure 9h), which enabled the demonstration of high-density cobalt single atoms within a graphene network rich in pyridinic nitrogen. The highly porous carbon network, along with increased conjugation and modification of adjacent cobalt sites, significantly enhanced its electrocatalytic performance in the OER in 1 M KOH (Figure 9a,b), achieving stability exceeding 300 h (Figure 9g). Through the synergistic metal–support interaction, the catalyst achieved a mass activity as high as 2209 mA mgCo^−1^/turnover frequency (TOF) at 1.65 V/0.37 s^−1^ (Figure 9c). Operando X-ray absorption near-edge structure (XANES) analysis revealed the formation of an electron-deficient Co-O coordination intermediate state, which accelerated the OER kinetics. Additionally, Wei Chen et al. [97] synthesized silver-loaded nitrogen-doped graphene sheets (Ag-NGs) nanocomposites using microwave plasma technology (Figure 9i). X-ray photoelectron spectroscopy (XPS) analysis indicated that silver nanoparticles preferentially adsorbed onto the graphite–nitrogen site structures, thus enhancing catalytic activity for the ORR. Moreover, a high level of nitrogen doping contributed to further improving the catalyst’s performance. This material exhibited excellent catalytic activity and remarkable stability in the four-electron pathway of the ORR, highlighting its potential as a promising electrocatalyst in clean energy applications.

Despite its vast application prospects in electrocatalysis, the catalytic activity of graphene still lags behind that of metal nanocatalysts. To effectively enhance the catalytic performance of graphene, heteroatom doping has proven to be an extremely effective strategy [98], significantly altering its electronic properties and thereby boosting its catalytic activity. For instance, Daniel San Roman et al. [99] introduced a new graphene-based hybrid nanomaterial called nanowire-templated out-of-plane three-dimensional fuzzy graphene (NT-3DFG). Utilizing adjustable synthesis methods, NT-3DFG demonstrated impressive efficiency, achieving an onset potential of 0.79 ± 0.01 V, high selectivity of 94 ± 2% for H_2_O_2_, and tunable ORR activity depending on the density of graphene edge sites. Zhang et al. [100] investigated the non-free radical pathways and active sites of nitrogen-doped graphene (N-rGO) activated by peroxymonosulfate (PMS). The introduction of nitrogen atoms into the graphene structure activated the sp^2^-hybridized carbon lattice, resulting in an abundance of free π electrons that enhanced catalytic activity. Within 10 min, the NH_3_-rGO-10/PMS system achieved a sulfamethoxazole (SMX) degradation rate of 93.66%, significantly higher than the 75.34% observed for non-radical pathways. Furthermore, even in the presence of various anions and differing concentrations of humic acid, the SMX removal rate consistently remained above 90%.

**Figure 10:**
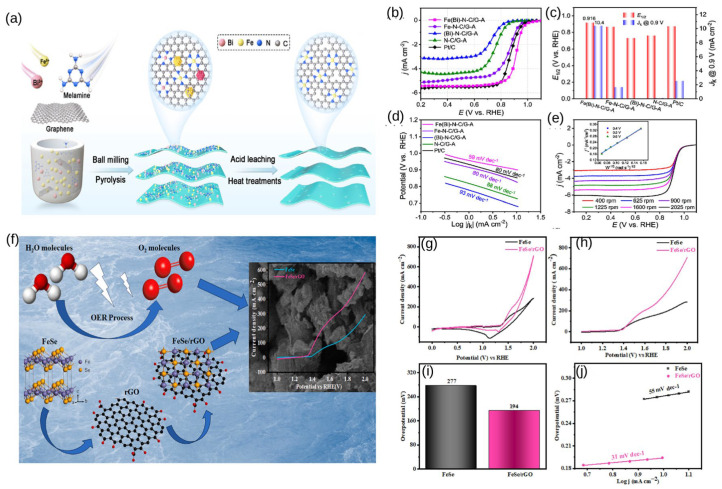
(**a**) Schematic illustration of the synthesis of dense Fe-N-C sites anchored onto graphene. (**b**–**e**) Electrochemical ORR activity of as-prepared samples. LSV curves (**b**) and J_k_ (at 0.9 V) and E_1/2_ (**c**). (**d**) Corresponding Tafel profile. (**e**) Polarization curves for Fe (Bi)-N-C/G-A at different rotation speeds (inset: K-L plot for Fe (Bi)-N-C/G-A) [104]. (**f**) Application of iron oxide–graphene composites in electrochemical catalytic reactions. (**g**) CV graph for FeSe and FeSe/rGO. (**h**) LSV graph for FeSe and FeSe/rGO. (**i**) Overpotential graph for FeSe and FeSe/rGO. (**j**) Tafel slope graph for FeSe and FeSe/rGO [105].

In addition to serving as substrates and active materials, graphene is widely used as a multifunctional carrier for the synthesis of strongly coupled metal-based electrocatalysts due to its high conductivity and tunable nanostructure [101]. For instance, Ma et al. [102] developed a novel single-atom functional Janus hollow graphene structure, consisting of two types of single atoms (Ni-N_4_ and Fe-N_4_) separated by a graphene layer (Ni-N_4_/GHSs/Fe-N_4_). This Janus material demonstrated exceptional bifunctional electrocatalytic performance, with the outer Fe-N_4_ clusters significantly enhancing activity for the ORR, while the inner Ni-N₄ clusters provided excellent activity for the OER. Furthermore, Lyu et al. [103] created nickel–iron nanoparticle nests supported on graphene (NiFe NNG). The addition of the graphene support improved the catalytic activity, electron transfer, and electrical conductivity of the NiFe-based catalyst. The NiFe NNG demonstrated outstanding OER performance in alkaline media, achieving a low overpotential of 292.3 mV at a current density of 10 mA cm⁻^2^, along with a small Tafel slope of 48 mV dec⁻¹. Yang et al. [104] presented a method for generating accessible Fe-N-C active sites supported on graphene by incorporating a removable bismuth compound. This approach effectively inhibited the formation of iron-related particles and tubular carbon structures, thereby enhancing the availability of active sites for catalytic applications (Figure 10a). The Fe(Bi)-N-C/G-A demonstrated outstanding ORR performance in alkaline (E_1/2_ of 0.916 V) and acidic (E_1/2_ of 0.784 V) environments, attributed to the unique structure obtained through a simple annealing process. It also exhibited remarkable durability, with minimal loss after 10,000 cycles and a high peak power density of 201.4 mW cm^−2^ for zinc–air batteries (Figure 10b–e). However, the scalability and reproducibility of these synthesis methods remain challenging, particularly for complex structures like Janus materials and Fe(Bi)-N-C systems. Furthermore, the long-term stability and performance of these catalysts under real-world operating conditions require further investigation to assess their practical applicability. Hu et al. [105] prepared a FeSe/reduced graphene oxide composite (FeSe/rGO) via a hydrothermal method (Figure 10f), employing it as an electrocatalyst for the OER. Compared to the benchmark commercial RuO_2_, the FeSe/rGO nanocomposite exhibited a minimal overpotential of 194 mV at a current density of 10 mA cm^−2^ for the OER and a small Tafel slope of 31 mV dec^−1^ (Figure 10g–j). Moreover, FeSe/rGO exhibited excellent stability and long-term durability. Calculations and experimental results demonstrated that the excellent catalytic performance was attributed to the synergistic effect between rGO and FeSe, enhancing the material’s electrical conductivity, specific surface area, and active sites, while promoting ion transport.

Despite the outstanding conductivity and electron transfer ability of graphene, its intrinsic catalytic activity remains relatively limited. Moreover, graphene’s performance can be adversely affected by the reaction environment, leading to structural damage or degradation [106]. For example, durability and cycling performance can be significantly compromised by Ostwald ripening of Pt and corrosion of the graphene support during the ORR [107]. Moreover, in the catalytic ozonation reaction for the degradation of oxalic acid, as the ozone treatment time prolongs, the acidity on the surface of graphene gradually increases and its catalytic activity gradually decreases. When graphene is used in some electrochemical reactions involving metal ions, the metal ions may interact with graphene, and then a metal–graphene complex may be formed. The formation of this complex can alter the electronic structure and surface properties of graphene, affecting its catalytic performance in electrochemical reactions and increasing the charge transfer resistance. Therefore, developing graphene-based catalysts with higher catalytic activity, improved stability, and lower costs is of crucial importance.

## 3. Conclusions

With the ever-increasing global energy demand and escalating environmental pollution issues, the development of efficient energy storage and conversion technologies has become crucial for addressing these challenges. Oxygen electrocatalysis, encompassing the oxygen evolution reaction (OER) and the oxygen reduction reaction (ORR), plays a pivotal role in advanced energy storage and conversion systems. Low-dimensional carbon nanomaterials, due to their unique physical and chemical properties, exhibit tremendous potential for applications in electrocatalysis. Table 2 and Table 3 summarize the ORR and OER activities of recently reported electrocatalysts based on low-dimensional carbon nanomaterials. CDs offer unique nano-size and quantum effects to induce the construction of effective electrocatalysts for the OER/ORR, but they generally exhibit lower conductivity than CNTs and graphene, requiring further research to enhance their stability. Meanwhile, the synthesis of CDs presents scalability challenges and inconsistencies in size and surface functionalization. CNTs are known for their exceptional mechanical strength and good conductivity, making them suitable for applications in oxygen electrocatalysis; however, their synthesis methods can be complex and energy-intensive, and issues related to the alignment and purity of CNTs can affect their performance. Furthermore, while metal-free-doped CNTs or metal-doped CNTs can improve their electrocatalytic properties, the process often lacks reproducibility and can lead to unwanted side reactions that hinder overall performance. Graphene is widely utilized to engineer remarkable catalysts in the field of oxygen electrocatalysis due to its excellent electrical conductivity and large surface area, although challenges remain in achieving uniform dispersion and managing production costs.

Accordingly, current research still faces several challenges, including optimization and exposure of active sites, catalyst stability, and scalable production. Firstly, the optimization and exposure of active sites remain challenging, as complex structures may hinder the full utilization of these active sites. Additionally, the stability of these nanomaterials, particularly under long-term electrochemical conditions, is a critical issue. For instance, graphene and CNTs can suffer from chemical corrosion or structural degradation, leading to reduced catalytic performance. On the other hand, scaling up the production of these nanomaterials is also difficult, with current synthesis methods often limited to laboratory settings, restricting their widespread application and increasing costs. Future research should focus on optimizing active sites, enhancing material stability, and improving scalability while mitigating environmental and health risks to ensure the safe and effective application of low-dimensional carbon nanomaterials in oxygen electrocatalysis.

**(1) Optimization of active sites:** Optimizing active sites in low-dimensional carbon materials is crucial for their effectiveness as oxygen electrocatalysts. One effective approach is to maximize the exposure of active sites by precisely controlling the morphology of the materials during synthesis. For example, tuning growth conditions can yield nanostructures with a high surface-to-volume ratio, such as nanotubes, nanosheets, or nanoparticles, which provide a larger number of surface atoms that can serve as potential active sites. Furthermore, enhancing catalytic efficiency is another key aspect of active site optimization. This can be achieved by regulating the structure and composition of the materials. Doping with heteroatoms, such as nitrogen, sulfur, or phosphorus, can modify the electronic structure of the carbon matrix, creating defects or introducing new active sites that facilitate the ORR and OER. Additionally, combining low-dimensional carbon materials with other functional components, such as metal nanoparticles or metal oxides, can form hybrid catalysts that exhibit synergistic effects, further improving catalytic activity and stability.

**(2) Enhancement of catalyst stability:** While low-dimensional carbon materials are promising oxygen electrocatalysts, their stability during long-term reactions presents a challenge. Two main strategies can be employed to address this: surface modification and structural design. Surface modification involves functionalizing the material’s surface, such as applying protective coatings (e.g., atomic layer deposition (ALD) of metal oxides like TiO_2_ and Al_2_O_3_) to shield active sites from chemical attacks. Additionally, element doping (e.g., boron, phosphorus) can enhance surface stability by altering surface energy and reactivity. Structural design is also crucial for stability; controlling porosity and creating structures (e.g., 3D network architectures) can optimize mass transport and mechanical stability. Core–shell structures, where a stable shell protects a catalytic core, have proven effective in reducing degradation. Overall, these strategies can significantly improve the stability of low-dimensional carbon-based oxygen electrocatalysts for practical applications in electrochemical devices.

**(3) Development of scalable production techniques:** The potential of low-dimensional carbon nanomaterials as oxygen electrocatalysts is contingent upon the development of scalable production techniques. Currently, researchers are exploring eco-friendly and efficient preparation methods. One promising approach is utilizing biomass and petroleum/coal-derived precursors for synthesizing carbon nanomaterials. Biomass and petroleum/coal byproducts are abundant, renewable, and cost-effective; by pyrolyzing biomass-based substances under controlled conditions, carbon nanomaterials with desirable properties can be produced. Another avenue is optimizing CVD techniques. By precisely controlling synthesis parameters, researchers can improve the growth rate and quality of carbon nanomaterials, facilitating large-scale production. Additionally, template-assisted synthesis methods are being investigated to produce carbon nanomaterials with uniform morphology and high-performance catalytic activities, which are crucial for their scalable application in oxygen electrocatalysis.

## Figures and Tables

**Table 1:** Synthesis methods, percentages, and their advantages and disadvantages for low-dimensional carbon nanomaterials.

Synthesis Method	Usage	Advantages	Disadvantages
Chemical Vapor Deposition (CVD)	42%	High-quality, high-purity carbon nanotubes and graphene. Strong controllability, suitablefor large-scale production. Adjustable growth rate and morphology.	High equipment requirements and cost. High reaction temperature, high energy consumption. Requires catalysts, which may affect material properties.
Liquid Phase Methods (Solvothermal, Liquid-PhaseReduction)	28%	Mild reaction conditions, easy to control. Suitable for large-scaleproduction, low equipment requirements. Can precisely control nanostructures by adjusting reaction conditions.	Longer synthesis time. Purity of products may belimited, requiring further purification. Selection of solvents andreactants is critical.
Laser-Enhanced Chemica Vapor Deposition (LCVD)	10%	High-quality carbon material scan be obtained. Laser enhances reaction rate improving production efficiency.	Complex and expensive equipment. High requirements forreaction conditions, difficult to operate.
Laser Ablation	5%	High-purity carbon nanomaterials. No catalysts, avoiding catalyst contamination.	Expensive equipment, high energy consumption. Low yield, not suitable for large-scale production. Difficult to control the synthesis process.
Arc Discharge	5%	High-purity carbonnanotubes, etc., can be synthesized. No catalysts required.	Expensive equipment, complex operation. Low yield, suitable forlaboratory-scale production. Requires high reaction environment control.
Microwave lrradiation	5%	Uniform heating, fast reactionspeed. Simple and easy to operate, compact equipment.	Expensive microwave equipment. High requirements for reaction conditions, prone to local overheating.
Self-Assembly	5%	Simple method, mild reactionconditions. Can achieve ordered carbonnanostructures.	Sensitive to solvent, pH, etc.challenging to control. Suitable for small-scale sample preparation.

**Table 2:** OER activities of CDs, CNTs, and graphene-based electrocatalysts.

Electrocatalyst	E10 (V vs. RHE)	Tafel Slope (mV dec^−1^)	Electrolyte	Reference
	OER	OER		
FeNi_3_@GCDs-10	238 mV	48.7	0.1 M KOH	[30]
NiO-Mn_2_O_3_-CDs	298 mV	141.1	0.1 M KOH	[34]
Co/N-CNT	390 mV	78.0	0.1 M KOH	[64]
CNT-550-NS	1610 mV	105.0	0.1 M KOH	[69]
Fe_25_−NZ8@Co_500_-CN	350 mV	82.2	0.1 M KOH	[70]
CoFe-Co_3_C@NCNTs-20	320 mV	121.5	0.1 M KOH	[71]
Co@H-NCNT	580 mV	57.6	0.1 M KOH	[73]
CoFe@NCNT	340 mV	146.3	0.1 M KOH	[74]
CoMM	351 mV		1 M KOH	[96]
NiFe NNG	292 mV	48.0	1 M KOH	[103]
Cd-MOF 1	279 mV	85.1	0.1 M KOH	[108]
BIF-90	460 mV		0.1 M KOH	[109]
MOFs 4	381 mV	75.1	0.1 M KOH	[110]
MOFs 5	397 mV	74.4	0.1 M KOH	[110]
BMMOF11	400 mV	85.0	0.1 M KOH	[111]
Co@C_1_N_3_C	625 mV	95.8	0.1 M KOH	[112]
Co/Co_3_Fe_7_@NCNTs-800	280 mV	86.0	0.1 M KOH	[113]
CoFe/S-N-C	1588 mV	259.0	0.1 M KOH	[114]
Co/SP-NC		85.0		[115]
CNT@Co_2_-Fe_1_/FePc		43.6	0.1 M KOH	[116]
FeNi-rGO/FeNi/Ni foam	234 mV	76.0	0.1 M KOH	[117]
NiFL	292 mV	45.4	1 M KOH	[118]
GQDs-Co_3_O_4_	321 mV	76.0	1 M KOH	[119]
ZFO-NG	240 mV	63.5	1 M KOH	[120]

**Table 3:** ORR activities of CDs, CNTs, and graphene-based electrocatalysts.

Electrocatalyst	E_1/2_ (V vs. RHE)	Tafel Slope (mV dec^−1^)	Electrolyte	Reference
	ORR	ORR		
S,N-GQD/TiO_2_/C-800	820 mV	61	0.1 M KOH	[28]
GP high N	685 mV		0.1 M KOH	[29]
BN-CDs@CNT	800 mV	72	0.1 M KOH	[32]
3%-N,P-PCNFs-900	720 mV	91	0.1 M KOH	[33]
NiO-Mn_2_O_3_-CDs	840 mV	126	0.1 M KOH	[34]
Co/N-CNT	850 mV	72	0.1 M KOH	[64]
Co@N-CNTs/3DHC	880 mV	45.6	0.1 M KOH	[67]
Fe_25_−NZ8@Co_500_-CN	850 mV	97.5	0.1 M KOH	[70]
CoFe-Co_3_C@NCNTs-20	934 mV	66	1 M KOH	[71]
CoFe@NCNT	840 mV	90.3	1 M KOH	[74]
GFePc	470 mV		pH=0	[94]
Vac-NiPt NPs/NG	901mV	58	0.1 M HClO_4_	[95]
CoMM		84	1 M KOH	[96]
Ag-NGs	820 mV	75.15	0.1 M KOH	[97]
NT-3DFG	830 mV	55	0.1 M KOH	[99]
Ni-N_4_/GHSs/Fe-N_4_	830 mV	81	0.1 M KOH	[102]
BIF-90	650 mV		0.1 M KOH	[109]
F, N-CD@Ag-0.1	900 mV	69	0.1 M KOH	[121]
TOCNFs	840 mV	84	0.1 M KOH	[122]
Co@C_1_N_3_C	840 mV		0.1 M KOH	[112]
Co/Co_3_Fe_7_@NCNTs-800	890 mV	107	0.1 M KOH	[113]
Co@N_4_CNTs			0.1 M KOH	[123]
CoFe/S-N-C	855 mV	102	0.1 M KOH	[114]
Co/SP-NC	860.3 mV	78	0.1 M KOH	[115]
Ni-N_4_/GHSs/Fe-N_4_	830 mV	81	0.1 M KOH	[116]
Pt/TaON/GLC	940 mV	62	0.1 M KOH	[124]
Pd/GO	810 mV	56	0.1 M KOH	[125]
PNG	800 mV		0.1 M KOH	[126]
N-rGO-CuSn			0.1 M KOH	[127]

## Data Availability

Data are contained within the article.
